# Biofortification of Cereals With Foliar Selenium and Iodine Could Reduce Hypothyroidism

**DOI:** 10.3389/fpls.2018.00730

**Published:** 2018-06-08

**Authors:** Graham Lyons

**Affiliations:** School of Agriculture, Food and Wine, University of Adelaide, Glen Osmond, SA, Australia

**Keywords:** biofortification, cereals, deficiency, hypothyroidism, iodine, iodine deficiency disorders (IDD), selenium, wheat

## Abstract

Concurrent selenium and iodine deficiencies are widespread, in both developing and developed countries. Salt iodisation is insufficient to ensure global iodine adequacy, with an estimated one-third of humanity at risk of hypothyroidism and associated iodine deficiency disorders (IDD). Agronomic biofortification of food crops, especially staples such as cereals, which are consumed widely, may be an effective component of a food system strategy to reduce selenium and iodine malnutrition. Iodine and selenium are needed in the optimum intake range for thyroid health, hence joint biofortification makes sense for areas deficient in both. Foliar application is recommended as the most effective, efficient, least wasteful method for selenium and iodine biofortification. Currently, selenium is easier to increase in grain, fruit, and storage roots by this method, being more phloem mobile than iodine. Nevertheless, strategic timing (around heading is usually best), use of surfactants and co-application with potassium nitrate can increase the effectiveness of foliar iodine biofortification. More research is needed on iodine transporters and iodine volatilisation in plants, bioavailability of iodine in biofortified plant products, and roles for nano selenium and iodine in biofortification. For adoption, farmers need an incentive such as access to a premium functional food market, a subsidy or increased grain yield resulting from possible synergies with co-applied fertilisers, enhancers, fungicides, and insecticides. Further research is needed to inform these aspects of foliar agronomic biofortification.

## Introduction

Malnutrition is the main cause of global human mortality, with over 50% of deaths attributed to diet-related diseases. Micronutrient deficiencies, notably iron (Fe), zinc (Zn), selenium (Se), iodine (I), and certain vitamins are widespread globally, affecting about 60% of the world’s population, and in many areas multiple deficiencies occur (Lyons and Cakmak, 2012). Dysfunctional food systems fail to provide optimum nutrition to populations, especially to vulnerable sub-groups such as infants, children, and pregnant and nursing women (White and Broadley, 2009). This has been exacerbated by high-yielding Green Revolution cereal varieties with grain often less micronutrient-dense than previously (Smolen et al., 2016a).

Biofortification of staple crops to achieve higher micronutrient concentrations in edible parts represents a *food system* strategy to address dietary deficiencies, with the potential to reach the neediest of the population (Haug et al., 2007; Bouis and Welch, 2010; Lyons and Cakmak, 2012). This approach, which links a nutritious agriculture with human health, can be more effective and sustainable than provision of food supplements (Lyons, 2014).

Previous research suggests that genetic biofortification (plant breeding and genetic engineering) may be more suitable for increasing pro-vitamin A carotenoids and Fe, whereas an agronomic (fertiliser) strategy may be more effective for Zn, Se, and I (Genc et al., 2004; Cakmak, 2008; Bouis and Welch, 2010; Lyons and Cakmak, 2012). Transgenics may play an important role in micronutrient biofortification (White and Broadley, 2009), as shown by the high-Fe variant of the popular IR64 rice variety (Trijatmiko et al., 2016). Biofortification using conventional breeding or transgenics is a long-term process. Furthermore, the success of genetic biofortification of Se and I depends largely on their plant available concentrations in the soil solution. In most soils, plant available Se, for example, comprises only about 2.5% of total Se (Tan et al., 2002). Agronomic and genetic biofortification are hence complementary (White and Broadley, 2009; Lyons and Cakmak, 2012).

If minerals such as Fe, Zn, Se, and I can be increased in staple foods, population status of these minerals can be increased without behavioural change (Bouis and Welch, 2010). Hence widely consumed cereals, especially wheat, provide a suitable vehicle for increasing population Se status using agronomic biofortification (Broadley et al., 2006; White and Broadley, 2009; Fairweather-Tait et al., 2011).

The iodothyronine deiodinases D1, D2, and D3, which are selenoenzymes, control thyroid hormone turnover and hence are crucial in thyroid gland metabolism. Selenium supply is prioritised to the thyroid under conditions of Se restriction. Concurrent deficiencies of Se and I may exacerbate hypothyroidism (Schomburg and Kohrle, 2008; Fairweather-Tait et al., 2011; Kohrle, 2013; Gashu et al., 2016), and low Se status increases risk of goitre, especially in women (Rasmussen et al., 2011; Schomburg, 2012; Wu et al., 2015). The more severe the Se deficiency, the less effective is I supplementation in alleviating goitre (Zimmermann et al., 2000; Drutel et al., 2013; O’Kane et al., 2018). Moreover, Se-dependent glutathione peroxidases protect the thyroid against oxidative stress, for example, due to excess I (Schomburg and Kohrle, 2008; Schomburg, 2012; Drutel et al., 2013; Kohrle, 2013).

Hypothyroidism is not the only pathological condition that can be exacerbated by concurrent I and Se deficiencies: myxoedematous cretinism, whose aetiology requires I and Se deficiency accompanied by a goitrogen (for example, TGF-beta, thiocyanates from cassava, *Fusarium* toxins in wheat), exists in parts of Tibet and the Democratic Republic of Congo (Contempre et al., 1992; Schomburg and Kohrle, 2008; Christophersen et al., 2012; Kohrle, 2013). In myxoedematous cretinism, hypothyroidism persists despite I supplementation (Contempre et al., 1992). Where both deficiencies occur, it is important to normalise I intake and status first, before supplementing with Se. If Se is supplemented first, hypothyroidism can worsen in the short term (Contempre et al., 1992).

**FIGURE 1:**
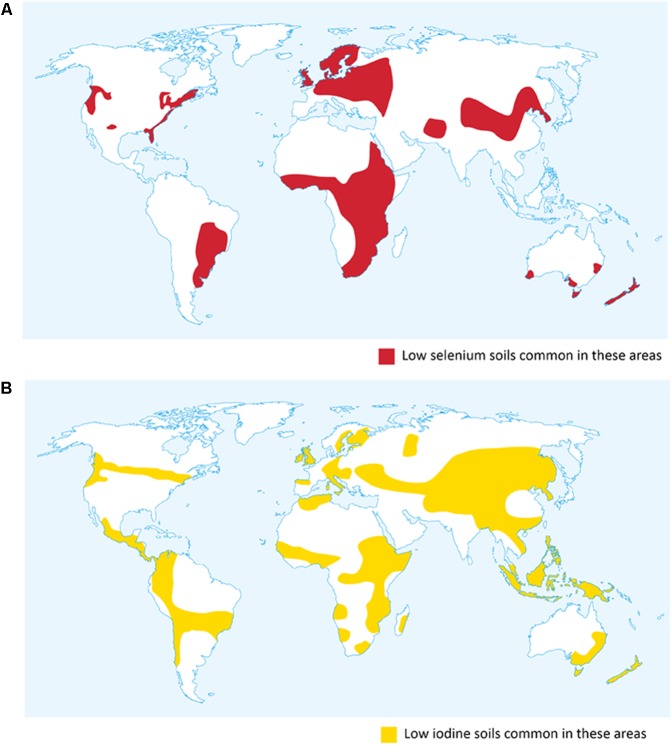
Global distribution of soils low in selenium and iodine. **(A)** Selenium distribution in the United States adapted from Oldfield (1999). **(B)** Iodine map adapted from Hetzel and Pandav (1994).

This mini-review will focus on research on agronomic biofortification of cereals with Se and I, and explore the proposal that simultaneous application of these micronutrients has the potential to reduce hypothyroidism and related iodine deficiency disorders (IDD) in areas with concurrent Se and I deficiencies (**Figure 1**).

## Selenium

### Profound Influence on Human Health With a Variable Distribution

The importance of Se to human health, in terms of its key roles in the thyroid, brain, heart, and gonads, along with heavy metal-binding, antioxidant, anti-cancer, anti-bacterial, and anti-viral activity, is indicated by its status as the only micronutrient to be specified in the human genome, as selenocysteine, the twenty-first amino acid (Rayman, 2000, 2002). Its deficiency is also linked to several diseases, including the osteoarthropathy, Kashin-Beck disease (KBD), which is still prevalent in parts of China, including the Loess Plateau in Shaanxi Province, and Tibet. Aetiological factors for KBD include *Fusarium* mycotoxins in infected grain, organic acids in drinking water, low dietary Se, and gene polymorphisms (Fairweather-Tait et al., 2011; Bissardon et al., 2017).

Although much less common than Se deficiency, Se toxicity can occur, for example in Enshi in the Chinese province of Hubei, when selenosis, characterised by hair loss and thickened nails, occurred, particularly from 1961 to 1964. It was caused by eating crops grown on high-Se soil (Yang et al., 1983). Daily recommended intake of Se is mostly 40–75 μg/day globally, with <30 μg/day inadequate and >900 μg/day potentially harmful; however, tolerable upper limits have been set lower, in the range of 400–450 μg/day for the United Kingdom, United States, Canada, EU, Australia, and New Zealand (Fairweather-Tait et al., 2011). Selenium’s interplay with human physiology is complex and integral, and deficiency, sufficiency, and toxicity span a relatively narrow range of Se intake and status (Vinceti et al., 2009; Fairweather-Tait et al., 2011; Winkel et al., 2012).

Selenium delivery in a food system depends mainly on the levels of plant available Se in soils used for agriculture. Selenium is ubiquitous but of uneven plant-availability, hence its variability in populations and their sub-groups. It is estimated that up to a billion people are deficient in Se (Combs, 2001; Haug et al., 2007; Winkel et al., 2012; Ros et al., 2016). The element’s availability in soils depends on soil pH, redox potential, cation exchange capacity, and levels of Fe, sulphur, aluminium, and carbon (Broadley et al., 2006; Chilimba et al., 2011; Christophersen et al., 2012; Winkel et al., 2012).

### Agronomic Biofortification: Foliar Selenate More Efficient

Selenium is well suited to agronomic biofortification of food crops. In the selenate form, it is readily taken up by plants growing on most soils, then transported throughout the plant, accumulating in edible parts. In cereals, it is converted mostly into selenomethionine, which is well represented in grain endosperm, hence Se can be abundant and bioavailable in milled products such as white flour and polished rice (Lyons and Cakmak, 2012).

Selenium form is important for effective biofortification. Most studies have shown selenate (where Se exists in its highest oxidation state, +6) to be easily the most effective form when applied to the soil and usually more effective than selenite (Se +4) when applied as a foliar (Broadley et al., 2006; Boldrin et al., 2013; Mao et al., 2014; Ros et al., 2016; Xiong et al., 2018). A recent review found selenate to be 33 times more effective overall than selenite (Ros et al., 2016). In many soils, selenite is rapidly adsorbed on clay colloids, rendering it poorly available to plants. Dry climate, low organic matter, high temperature, high soil pH, and aeration are likely to increase the selenate: selenite ratio in the soil and hence the availability of Se to plants (Christophersen et al., 2012).

In Finland, the use of Se (selenate) fertilisers commenced on a national scale in 1984, resulting in a fourfold increase in dietary Se intake and doubling of the plasma/serum Se concentrations of the study population. There were concerns that the addition of Se in this manner may have long-term environmental effects. In California, for example, drainage water collected from an irrigated area overlaying a high-Se shale resulted in deaths and malformations in fish and aquatic birds at the Kesterson reservoir in the 1980s (Hartikainen, 2005). A study of lake and ground water in Finland in 1992 found no differences in Se concentration in water from lakes in agricultural and non-agricultural areas. Ground water samples were variable in Se (33–260 μg/l), partly explained by different Se concentrations of bedrock and sediments, and some leaching from fertilisers (as indicated by correlations with phosphorus and nitrogen) in certain areas (Mäkelä et al., 1995). The ongoing Finnish biofortification programme demonstrates the relative safety, effectiveness, ease, and cost-efficiency of this strategy (Eurola et al., 1990; Broadley et al., 2006; Haug et al., 2007; Winkel et al., 2012; Ros et al., 2016). This model could be applied to other low-Se countries, like Malawi (Chilimba et al., 2012).

Nevertheless, Se soil biofortification is a relatively wasteful process. The recovery of soil-applied Se in wheat grain varies from 5 to 32%, with an estimated average of about 12% (Eich-Greatorex et al., 2007; Haug et al., 2007; Broadley et al., 2010; Lyons and Cakmak, 2012; Ros et al., 2016). Selenium is a valuable, mostly non-renewable resource, which should be conserved (Haug et al., 2007; Ros et al., 2016).

Foliar application has usually been found to be more efficient than soil application for Se (Ylaranta, 1984; Mao et al., 2014; Winkel et al., 2015; Ros et al., 2016; Gupta and Gupta, 2017). Foliar application not only obviates the soil factors that can reduce the effectiveness of soil agronomic biofortification of Se, but also reduces possible environmental Se accumulation (see above) as less Se is applied per hectare. Timing of foliar Se and I application is important, with the best effect usually obtained between booting and early milk stage, with heading, when green leaf cover is maximised, the “best bet” for an effective single application. A recent meta-analysis enables estimation of the amount of selenate needed to increase grain Se from 7 to 100 μg/kg: 30–60 g/ha soil-applied selenate, and 4.5–10 g/ha foliar selenate. This study found foliar-applied Se to be on average eight times more efficient than soil-applied Se (Ros et al., 2016). In tropical/sub-tropical countries where Se fertilisers are unavailable, leaves of the Drumstick tree (*Moringa oleifera*), which has exceptional ability to take up and accumulate Se, can provide useful levels of Se, even when grown on soils that provide little Se to most other plants (Lyons et al., 2015).

## Iodine

### Iodised Salt Needs Help to Fix Global Iodine Insufficiency

Iodine is essential to humans, being required for synthesis of thyroid hormones, which are essential for human development and health. Requirement is in the range 90–250 μg/day. Inadequate I is one of the major micronutrient deficiencies, leading to a range of clinical and social issues known as IDD. The classic symptom of I deficiency is an enlarged thyroid, known as goitre (Zimmermann et al., 2008). The safe upper limit of I intake is estimated at 1000–1100 μg/day; chronic intakes above this level can increase risk of Graves disease (Surks et al., 2004; Leung et al., 2015). Like Se, plant-available I is unevenly distributed (**Figure 1**): the sea is an important source, hence I in food systems usually declines with distance from it. Inland, high rainfall, mountainous areas are notoriously deficient in I. The overall global average soil I concentration is 2.6 mg/kg (Hetzel, 1989; Watts et al., 2010), but I concentration in plants grown on I-deficient soils may be as low as 10 μg/kg, compared with 1 mg/kg in plants on an I-replete soil (Hetzel and Pandav, 1994).

Although the number of countries designated as I deficient halved in the decade to 2014 (Gonzali et al., 2017), I deficiency remains prevalent, affecting an estimated 33% of humanity (Fuge and Johnson, 2015). Marginal I status is even present in developed countries, including England, Germany, Italy, and Australia (Andersson et al., 2012). It is apparent that iodisation of salt is insufficient to ensure overall I adequacy. Contributing factors include lack of availability of iodised salt for all households, food manufacturers not using iodised salt, volatilisation of I during food transport, storage, and cooking (on average, 20% of I in iodised salt is lost during cooking), and in many countries salt consumption has declined due to public health measures to reduce hypertension (Winger et al., 2008; White and Broadley, 2009; Comandini et al., 2013; Medrano-Macias et al., 2016; Smolen et al., 2016a; Cakmak et al., 2017). Most terrestrial foods are low in I. Strategies complementary to the iodised salt programme are needed, such as production of I-rich plants (White and Broadley, 2009; Comandini et al., 2013; Medrano-Macias et al., 2016; Smolen et al., 2016a; Cakmak et al., 2017). Vegetables biofortified with foliar I showed a high I stability during cooking (Comandini et al., 2013). Genetic approaches may be productive, for example *metabolic engineering* to reduce the problem of I volatilisation (Gonzali et al., 2017).

### Agronomic Biofortification: Foliar Iodate More Effective, but Easier to Biofortify Leaves Than Fruits, Roots, Grains, and Seeds

To address I insufficiency, researchers have urged the WHO to move beyond an iodised salt focus to a broader food system strategy that includes I biofortification of a range of vegetables (Smolen et al., 2016a). A case study of introducing I via agriculture was a spectacular success in Xinjiang province in north-west China. Potassium iodate was dripped into irrigation canals and resulted in a threefold increase in soil I levels, a twofold increase in I in wheat straw, increases in animal and poultry production, and in humans a 50% reduction in infant mortality and virtual elimination of IDD. Benefits were evident up to 7 years later (Jiang et al., 1997; Duxbury et al., 2015).

Most studies have shown that iodate is more suitable than iodide for biofortification (Mackowiak and Grossl, 1999; Dai et al., 2006 Lawson et al., 2015; Medrano-Macias et al., 2016; Smolen et al., 2016b; Cakmak et al., 2017). Iodate is also more likely than iodide to promote plant growth and less likely to be phytotoxic (Borst-Pauwels, 1961; Blasco et al., 2008). Iodide is more available than iodate in solution culture, while under field conditions it is more subject to cumulative losses (Lawson et al., 2015).

Iodine in plants, unlike Se, is transported mostly (but not entirely: see below) in xylem tissue (Mackowiak and Grossl, 1999), hence it is relatively easy to biofortify leaves, and thus leafy vegetables such as cabbage, lettuce, spinach (Smolen et al., 2014, 2016a,b). It is more difficult to increase I levels in grain or storage roots/tubers (Mackowiak and Grossl, 1999; Hurtevent et al., 2013; Mao et al., 2014; Medrano-Macias et al., 2016; Gonzali et al., 2017). Hence there are more published articles to date on I biofortification of vegetables than for cereals. These vegetable articles provide valuable knowledge of I behaviour in plants that can be applied to cereals.

### Evidence for Phloem Mobility Supports Iodine Biofortification for Cereals

A comprehensive study that included glasshouse and field trials of cereals (wheat, rice, maize) in Pakistan, Brazil, Thailand, and Turkey, showed that foliar-applied I can increase grain I (Cakmak et al., 2017). For example, in a pot trial, wheat grain I was increased from 21 to 296 μg/kg using two applications (at heading and early milk stage) of potassium iodate (0.065%) plus a non-ionic surfactant (0.05%) and potassium nitrate (1%). The surfactant and potassium nitrate had an additive effect in enhancing I biofortification. In a field trial in Brazil, potassium iodate (0.05%) applied twice increased grain I from 8 to 485 μg/kg. Other studies also found that surfactants increased the efficiency of foliar micronutrient biofortification (Lawson et al., 2015; Gonzali et al., 2017). Foliar I biofortification was most effective for wheat, followed by rice, then maize (Cakmak et al., 2017).

The study of Cakmak et al. (2017) adds to recent evidence of phloem mobility of I in wheat (Hurtevent et al., 2013) and vegetables (Kiferle et al., 2013; Smolen et al., 2014; Li et al., 2017). The mechanism of potassium nitrate’s enhancement of leaf absorption and possibly translocation to grain of I may relate to the chemical similarity of nitrate and iodate and is worthy of investigation (Cakmak et al., 2017).

## Biofortification of Cereals With Selenium and Iodine Could Reduce Iodine Deficiency Disorders

### Combined Selenium and Iodine Foliar Biofortification: A Promising Strategy for Many Areas

In the extensive parts of Sub Saharan Africa, China, South America, Europe, and New Zealand with concurrent Se and I deficiencies (**Figure 1**) (Hetzel and Pandav, 1994; Oldfield, 1999; Gashu et al., 2016), foliar agronomic biofortification with both Se and I may be effective in increasing the supply of both micronutrients in food systems (Smolen et al., 2016a). Resulting health benefits would be likely to include reduced incidence and prevalence of hypothyroidism with its consequent spectrum of IDD and myxoedematous cretinism.

The suitability of foliar Se application for cereal grain biofortification, irrespective of soil type, was discussed above, while the findings of Cakmak et al. (2017) for foliar I biofortification of cereals are promising. Given the observed enhancement of I biofortification provided by potassium nitrate, trials to assess its effect on Se foliar biofortification may also be fruitful.

In view of the optimum molar ratio of I:Se, which is in the range of 4.4–8.8:1 (with an average around 6) in the human diet, calculated from the RDIs of 150–250 μg/day for I and 55–65 μg/day for Se (Smolen et al., 2016a), plausible target levels of I and Se in cereal grain could be 1.0 and 0.25 mg/kg, respectively. Toxic effects can be expected at chronic Se intakes in livestock feed/human food that exceed 1 mg/kg (Hartikainen, 2005). There is an agreeable symmetry in a joint biofortification concept for Se and I, their importance for the thyroid notwithstanding, given their juxtaposition on the Periodic Table.

### Could Se+I Foliar Biofortification of Cereals Be Attractive to Farmers?

For agronomic biofortification to become commercial, it needs to benefit both producers and consumers (Bouis and Welch, 2010; Cakmak et al., 2010). Cereal yield is unlikely to be increased by Se and/or I application (Lyons and Cakmak, 2012; Ros et al., 2016), therefore fertiliser containing Se and I may need to be subsidised (Chilimba et al., 2012), or a biofortified product could attract a premium price as a desirable *functional food*.

Although considered to be non-essential to plants, Se and I can be beneficial. For example, Se addition increased biomass in mungbean (*Phaseolus aureus*) (Malik et al., 2010) and turnip (*Brassica rapa* var. *rapa*) (Xiong et al., 2018), increased seed production in canola (*Brassica rapa)* (Lyons et al., 2009), and improved quality and shelf-life of vegetables and fruits (Puccinelli et al., 2017). Selenate and selenite at low doses increased growth and sulphur accumulation in wheat seedlings, but these effects were not seen in grain (Boldrin et al., 2016). Iodine use in agriculture has been reviewed by Medrano-Macias et al. (2016). Iodine is involved in various plant physiological and biochemical processes (Gonzali et al., 2017). Benefits include growth enhancement, increased nitrogen uptake, increased sugars and amino acids, improved seed viability, and increased tolerance to salinity and heavy metals via induction of antioxidants including ascorbate, glutathione, and superoxide dismutase (Borst-Pauwels, 1961; Medrano-Macias et al., 2016; Gonzali et al., 2017).

Potential benefits from applying Se and I, including increased growth and product quality, together with the convenience and economy of combining them with strategic fertiliser, fungicide and insecticide applications, could make Se+I biofortification commercially viable for farmers.

### Further Research Needed

Research needed on combined Se+I biofortification includes evaluation of potential enhancers, including salicylic acid, a phytohormone-like compound, which improved tomato fruit biofortification with I (Smolen et al., 2015), the pineal gland hormone melatonin, which is also present in plants and can act as a synergist with antifungal agents (Zhang et al., 2017), the carrier dimethyl sulfoxide, which increased the effectiveness of foliar Fe application (Leonard, 2006) and synergists such as potassium nitrate (Cakmak et al., 2017). Trials of co-application with fungicides and insecticides are also recommended, due to promising earlier findings (Mahmoud et al., 1996; Costa et al., 2003; Zhang et al., 2003; Hanson et al., 2004). Knowledge of I transporters in plants is incomplete (White and Broadley, 2009; Gonzali et al., 2017), and such research could include I volatilisation studies (Gonzali et al., 2017). An efficient single application of Se+I will be more acceptable to farmers than multiple applications.

Nanotechnologies in agriculture are attracting interest (De Rosa et al., 2010; Liu and Lal, 2015). Bioavailable biogenic elemental Se (BioSe), for example, is widespread in the microbial environment (Winkel et al., 2012). For roles in foliar biofortification, Se and I nanoparticles need to be well characterised, including particle size: stomatal openings are about 20 nm in diameter, thus movement of particles larger than this is problematic (Alshaal and El-Ramady, 2017).

More bioavailability studies that examine losses of Se and I from biofortified cereals during milling and during various cooking methods are also required, along with speciation of I in biofortified cereals.

## Author Contributions

GL researched, wrote, and checked the manuscript.

## Conflict of Interest Statement

The author declares that the research was conducted in the absence of any commercial or financial relationships that could be construed as a potential conflict of interest.
